# The sociocultural effects on orthopedic surgeries in Taiwan

**DOI:** 10.1371/journal.pone.0195183

**Published:** 2018-03-29

**Authors:** Shin-Lin Chiu, Mei-Jih Gee, Chih-Hsin Muo, Chiao-Lee Chu, Shou-Jen Lan, Chiu-Liang Chen

**Affiliations:** 1 Department of Ophthalmology, Changhua Christian Hospital, Changhua City, Taiwan; 2 Department of Optometry, Da Yeh University, Changhua City, Taiwan; 3 Department of Statistics, Fong Chia University, Taichung City, Taiwan; 4 Management Office for Health Data, China Medical University Hospital, Taichung City, Taiwan; 5 Department of Long Term Care, National Quemoy University, Quemoy County, Taiwan; 6 Department of Healthcare Administration, Asia University, Taichung City, Taiwan; 7 Department of Medical Research, China Medical University Hospital, Taichung City, Taiwan; 8 Department of Orthopedics, Changhua Christian Hospital, Changhua City, Taiwan; 9 Department of Nursing, Da Yeh University, Changhua City, Taiwan; London Health Sciences Centre University Hospital, CANADA

## Abstract

Various sociocultural factors affect healthcare-seeking behaviors. In Taiwanese society, superstitions and lunar festivals play important roles in people’s lives. We investigated the impact of “Ghost Month” (the 7^th^ lunar month) and Chinese New Year (the 12^th^ lunar month and the 1^st^ lunar month of the following year) on the number of elective surgeries and emergent surgeries in Taiwan. The number of total knee replacement (TKR) surgeries and proximal femur fracture (PFF) surgeries in each lunar month from 2000 to 2011 were extracted from the Taiwan National Health Insurance Database, a computerized and population-based database. Patients were then sorted by location of residence or gender. The average number of TKR surgeries performed was significantly lower during the 1^st^, 7^th^, and 12^th^ lunar months in urban areas, whereas in rural areas this trend was only evident in the 7^th^ and 12^th^ lunar months. There was however, no significant difference in the average number of PFF surgeries in each lunar month except for an increase seen in the 1^st^ lunar month in rural patients (p<0.05). When sorted by gender, the average number of TKR surgeries was significantly decreased in the 7^th^ and 12^th^ lunar months in male patients, and decreased in the 1^st^, 7^th^, and 12^th^ lunar months in female patients. In contrast, there was no difference in the average numbers of PFF surgeries in the 7^th^ and 12^th^ lunar months either in male or female patients. We proposed that the timing of elective surgeries such as TKR might be influenced by Ghost Month and Chinese New Year; however, emergent PFF surgeries were not significantly influenced by sociocultural beliefs and taboos in Taiwan.

## Introduction

Different sociocultural factors have shown potential influence on health-seeking behaviors [1–3]. Many cultures believe in the existence of ghosts [4–6]; moreover, superstitious beliefs affect social and health-seeking behaviors in many countries [7, 8].

In Taiwanese culture, there are four main lunar events including the Chinese New Year, the Dragon Boat Festival, the Ghost Month, and the Moon festival. The Dragon Boat Festival falls on the 5^th^ day of the 5^th^ lunar month. The Moon festival is celebrated on the 15^th^ day of the 8^th^ lunar month. The Ghost Month is the 7^th^ lunar month, which has no national holidays. The celebration of the Chinese New Year usually starts from the 16^th^ day of the 12^th^ lunar month to the 15^th^ day of the 1^st^ lunar month of the following year, with only five national holidays in this time period.

Most Taiwanese avoid elective surgeries during the Chinese New Year festival. It results from the cultural belief that it is bad luck to begin a lunar New Year with an illness, especially if a surgery is involved. In addition, most Taiwanese consider that the Ghost Month is a month of mishaps [9, 10]. It is believed that during this month, the gates of hell are opened, and the ghosts from the underworld are able to return to the world of the living, with hungry and vicious ghosts spending the month seeking souls to take their place in the underworld. Taiwanese provide offerings to these wandering ghosts, so that they will not bring harm. The Ghost Month is deemed a dangerous time period of the year, filled with mishaps. Because of this belief, people avoid potentially dangerous activities such as participating in watersports, mountain climbing, undergoing surgery, etc. during this month.

In addition to sociocultural taboos stemming from the lunar calendar, we were also interested in the disparity between urban and rural locality, and in that between genders. The level of influence varies between urban and rural populations, as rural populations are generally considered more traditional. Like many cultures in Taiwan, there exists an inequality in social status between men and women, so this study also probed at the topic of whether these culture taboos have an impact on the number of surgeries in each gender.

To the best of our knowledge, there were very few studies which addressed the impact of sociocultural taboos on surgery. Our study attempted to determine the effect of these cultural beliefs on elective and emergent surgeries in Taiwan. We further investigated the impact of urban and rural locality and gender differences on these surgeries.

## Materials and methods

### Database

This study was conducted as a population-based retrospective review by using the Taiwan National Health Insurance Research Database (NHIRD), which is one of the largest and most comprehensive population-based health databases in the world [11]. The NHIRD contains data on registration files, diagnoses, treatments, medications, types of surgery, and reimbursement claims. These data files are de-identified by scrambling the identification codes of both patients and medical facilities [12]. Taiwan is located in the eastern part of Asia with a population of 23 million people [13]. The study used data from the Longitudinal Health Insurance Database 2000 (LHID 2000), which is a subset of the NHIRD. The LHID 2000 contains the complete original insurance claims data of one million insured individuals who are randomly selected from the NHIRD, which represents about 4.5% of Taiwanese population. There are no significant differences in age and sex between LHID and the original NHIRD [14]. We utilized the LHID2000 database to gather information on patients’ demographics including encrypted identification number, gender, age, date of surgery, surgery code, and patients’ location of residence.

### Study sample

We extracted sample data from LHID2000 from 2000 to 2011 (S1 Table). The surgery codes were according to the reimbursement codes from the bureau of National Health Insurance. The surgery codes in our study were as follows: 64164B for TKR; 64029B for open reduction with internal fixation for PFF. TKR surgeries were regarded as elective surgeries that could be scheduled according to patients’ or physicians’ preferences. On the other hand, PFF surgeries were defined as emergent surgeries. Since proximal femur fractures usually occur in elderly patients due to low-energy trauma, resulting in severe pain, inability to ambulate, and subsequent cardiac or pulmonary compromise from prolonged bed rest. PFF surgeries should be performed at the earliest possible time to reduce complications such as pressure sores, pneumonia, or urinary tract infections. As previously mentioned, there are four major lunar events in Taiwan, but the Dragon Boat Festival and the Moon festival are single day events, which are less likely to have an overall impact on the number of elective versus emergent surgeries. Therefore, we chose the 1st, 7th, and 12th lunar months (with the 1^st^ and 12^th^ lunar months representing the Chinese New Year and the 7^th^ representing Ghost Month) to investigate the influences of lunar calendar events on the number of TKR and PFF surgeries performed.

Because the aim of this study was to elucidate the sociocultural effects of the 1^st^, 7^th^, and 12^th^ lunar months on the number of surgery performed, all other months, including the 2^nd^, 3^rd^, 4^th^, 5^th^, 6^th^, 8^th^, 9^th^, 10^th^, and 11^th^ lunar months, were set as baseline for comparison. The average numbers of surgeries performed in the 1^st^, 7^th^, and 12^th^ lunar months were compared to the baseline, respectively.

An interesting characteristic of the Chinese lunar calendar is that it has intercalary months, meaning duplicate months in a given year. There were two 4^th^ lunar months, two 2^nd^ lunar months, two 7^th^ lunar months, and two 5^th^ lunar months in 2001, 2004, 2006, and 2009 respectively.

The 7^th^ lunar month is the Ghost Month in Chinese lunar calendar. The 7^th^ lunar month appears on a different date as compared with Gregorian calendar months. The corresponding Gregorian calendar dates of each lunar month in the years studied is illustrated in Table 1. The number of surgeries on intercalary month was calculated using the arithmetic mean of the two identical numeric lunar months.

**Table 1:** The corresponding Gregorian calendar dates of lunar months from 2000 to 2011.

	1^st^ lunar month	2^nd^ lunar month	3^rd^ lunar month	4^th^ lunar month	5^th^ lunar month	6^th^ lunar month	7^th^ lunar month	8^th^ lunar month	9^th^ lunar month	10^th^ lunar month	11^th^ lunar month	12^th^ lunar month
2000	2/5-3/5	3/6-4/4	4/5-5/3	5/4-6/1	6/2-7/1	7/2-7/30	7/31-8/28	8/29-9/27	9/28-10/26	10/27-11/25	11/26-12/25	12/26-1/23
2001	1/24-2/22	2/23-3/24	3/25-4/22	4/23-5/22 5/23-6/20	6/21-7/20	7/21-8/18	8/19-9/16	9/17-10/16	10/17-11/14	11/15-12/14	12/15-1/12	1/13-2/11
2002	2/12-3/13	3/14-4/12	4/13-5/11	5/12-6/10	6/11-7/9	7/10-8/8	8/9-9/6	9/7-10/5	10/6-11/4	11/5-12/3	12/4-1/2	1/3-1/31
2003	2/1-3/2	3/3-4/1	4/2-4/30	5/1-5/30	5/31-6/29	6/30-7/28	7/29-8/27	8/28-9/25	9/26-10/24	10/25-11/23	11/24-12/22	12/23-1/21
2004	1/22-2/19	2/20-3/20 3/21-4/18	4/19-5/18	5/19-6/17	6/18-7/16	7/17-8/15	8/16-9/13	9/14-10/13	10/14-11/11	11/12-12/11	12/12-1-9	1/10-2/8
2005	2/9-3/9	3/10-4/8	4/9-5/7	5/8-6/6	6/7-7/5	7/6-8/4	8/5-9/3	9/4-10/2	10/3-11/1	11/2-11/30	12/1-12/30	12/31-1/28
2006	1/29-2/27	2/28-3/28	3/29-4/27	4/28-5/26	5/27-6/25	6/26-7/24	7/25-8/23 8/24-9/21	9/22-10/21	10/22-11/20	11/21-12/19	12/20-1/18	1/19-2/17
2007	2/18-3/18	3/19-4/16	4/17-5/16	5/17-6/14	6/15-7/13	7/14-8/12	8/13-9/10	9/11-10/10	10/11-11/9	11/10-12/9	12/10-1/7	1/8-2/6
2008	2/7-3/7	3/8-4/5	4/6-5/4	5/5-6/3	6/4-7/2	7/3-7/31	8/1-8/30	8/31-9/28	9/29-10/28	10/29-11/27	11/28-12/26	12/27-1/25
2009	1/26-2/24	2/25-3/26	3/27-4/24	4/25-5/23	5/24-6/22 6/23-7/21	7/22-8/19	8/20-9/18	9/19-10/17	10/18-11/16	11/17-12/15	12/16-1/14	1/15-2/13
2010	2/14-3/15	3/16-4/13	4/14-5/13	5/14-6/11	6/12-7/11	7/12-8/9	8/10-9/7	9/8-10/7	10/8-11/5	11/6-12/5	12/6-1/3	1/4-2/2
2011	2/3-3/4	3/5-4/2	4/3-5/2	5/3-6/1	6/2-6/30	7/1-7/30	7/31-8/28	8/29-9/26	9/27-10/26	10/27-11/24	11/25-12/24	12/25-1/22

### Statistical analysis

The statistical analysis was performed by using the SPSS software (version 20, SPSS Inc., Chicago, Illinois, USA). We utilized the linear mixed model that the covariance structure was heterogeneous AR (1) to the longitudinal data for investigating the differences in the number of surgeries between the mentioned lunar month and other lunar months. As the numbers of surgeries in conjoint lunar months were correlated, and the variance in each lunar month may be different, the covariance was set as heterogeneous AR(1) in linear mixed model. We set the number of surgeries in the 2^nd^, 3^rd^, 4^th^, 5^th^, 6^th^, 8^th^, 9^th^, 10^th^, and 11^th^ lunar months as baseline to compare against 1^st^, 7^th^ and 12^th^ lunar months. The F statistic was used to test whether two or more means were equal, with statistical significance set at p<0.05.

### Ethics

We utilized the NHIRD which was available for academic investigation. This study adhered to strict confidentiality guidelines that were in accordance with the regulations regarding personal electronic data protection. Because the data files contained unidentified secondary data, the study was exempted from a full review by the institutional Review Board (IRB) of Changhua Christian Hospital (IRB No. 150615) and was conducted in accordance with the Declaration of Helsinki. As the data files were de-identified, informed consent from the study population was not required.

## Results

The descriptive statistics of the average number of TKR and PFF surgeries in each lunar month from 2000 to 2011 are shown in Table 2. In S2 Table, a statistically significant difference (p<0.05) was noted when number of TKR surgeries was compared with each other over 12-month period.

**Table 2:** Descriptive statistics of the average numbers for total knee replacement surgeries and proximal femur fracture surgeries.

Lunar month	Total knee replacement				Proximal femur fracture			
	Urban		Rural		Urban		Rural	
	Male	Female	Male	Female	Male	Female	Male	Female
**1^st^**	5.08(2.021)	15.08(5.160)	8.50(3.289)	18.08(5.316)	12.33(6.213)	12.42(5.869)	15.42(5.178)	13.67(3.651)
**2^nd^**	5.88(4.146)	19.50(7.550)	7.54(3.677)	21.67(9.109)	9.88(3.178)	12.17(5.024)	11.04(2.684)	11.33(3.939)
**3^rd^**	5.83(3.129)	19.67(8.049)	8.42(2.610)	25.50(8.285)	9.08(3.204)	9.33(2.774)	9.75(3.646)	9.83(4.387)
**4^th^**	5.62(4.354)	19.13(11.205)	6.46(3.882)	20.04(9.850)	9.83(3.326)	8.71(3.194)	8.37(3.379)	11.38(4.773)
**5^th^**	5.17(3.689)	18.29(9.896)	6.67(3.576)	21.13(8.718)	9.83(2.758)	9.42(2.575)	9.46(3.201)	10.96(3.019)
**6^th^**	6.25(3.194)	23.08(9.940)	7.50(3.606)	25.08(6.259)	8.33(2.535)	10.33(5.211)	10.25(3.494)	9.50(4.543)
**7^th^**	3.87(1.760)	10.50(4.852)	6.00(2.045)	13.29(6.475)	9.92(2.466)	10.46(3.461)	9.29(4.137)	11.46(2.965)
**8^th^**	6.25(3.166)	21.17(11.448)	7.67(3.473)	22.58(8.501)	9.58(1.832)	9.92(3.777)	10.08(3.397)	10.17(2.791)
**9^th^**	6.00(2.730)	17.58(6.317)	8.67(4.479)	21.08(7.077)	9.25(2.864)	8.75(4.093)	9.33(2.995)	11.50(4.503)
**10^th^**	5.67(2.535	18.33(7.253)	8.25(3.745)	20.25(6.122)	9.33(2.902)	9.58(2.746)	12.33(3.916)	12.92(3.777)
**11^th^**	5.00(2.296)	17.58(7.255)	6.33(3.525)	19.67(6.893)	9.25(2.417)	12.08(4.776)	10.83(3.326)	13.50(4.145)
**12^th^**	2.00(1.651)	7.58(4.926)	2.75(1.815)	5.67(2.934)	8.58(5.160)	7.92(4.889)	9.67(5.929)	10.50(5.885)
**Average**	**5.22(3.119)**	**17.29(8.874)**	**7.06(3.618)**	**19.50(8.763)**	**9.60(3.448)**	**10.09(4.246)**	**10.49(4.129)**	**11.39(4.168)**

All values are reported as mean (standard deviation).

**Fig 1:**
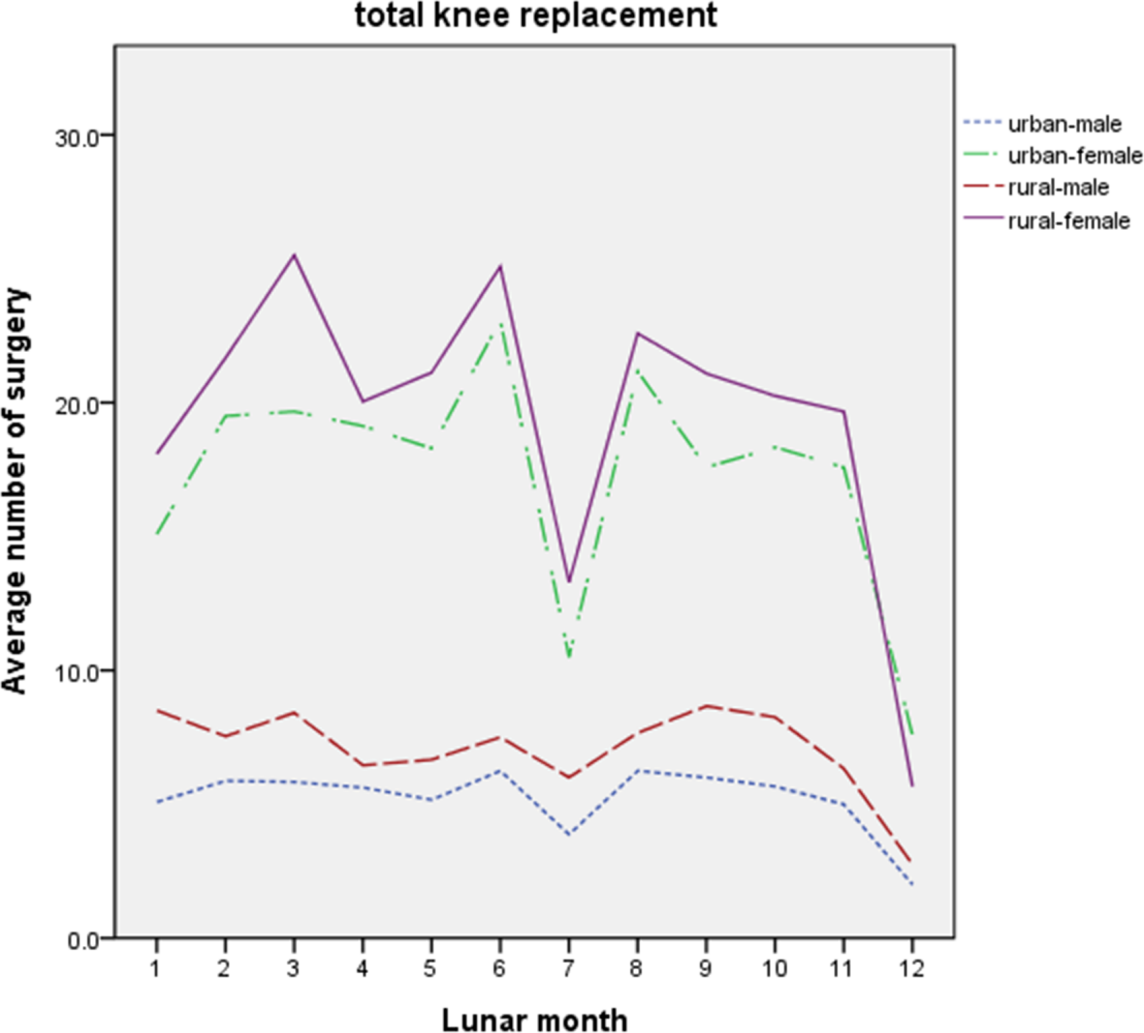
Average numbers of TKR surgeries categorized by location of residence and gender of patients in each lunar month from 2000 to 2011. X-axis: the 12 lunar months; Y-axis: the average number of the TKR surgeries. TKR: total knee replacement.

**Fig 2:**
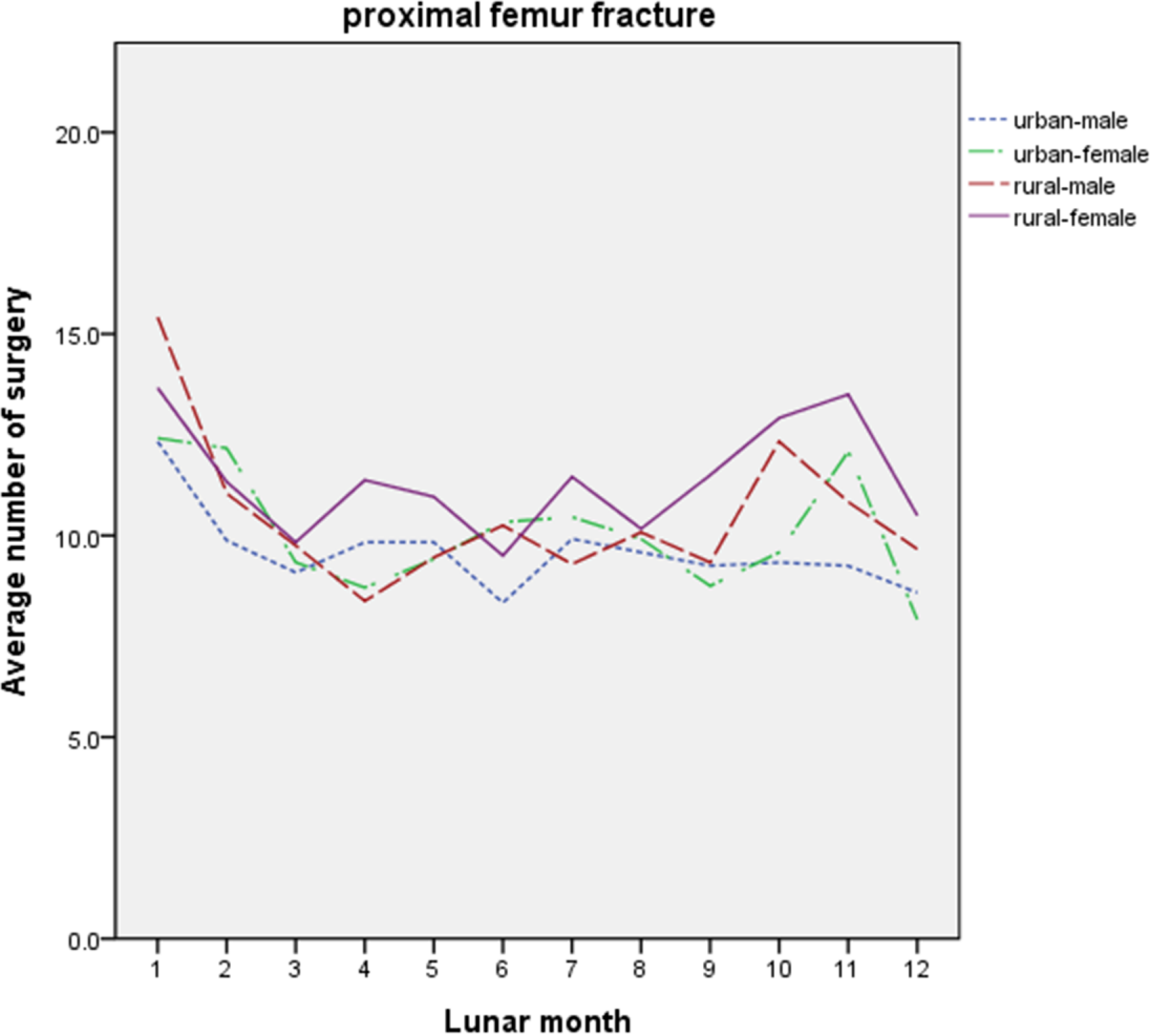
Average numbers of PFF surgeries categorized by location of residence and gender of patients in each lunar month from 2000 to 2011. X-axis: the 12 lunar months; Y-axis: the average number of the PFF surgeries. PFF: proximal femur fracture.

To investigate the influence of urban-rural gap and disparity between men and women, the patients were categorized according to the location of residence and gender. The average numbers of TKR surgeries and PFF surgeries categorized by location of residence and gender of patients in each lunar month from 2000 to 2011 are displayed in Figs 1 and 2, respectively.

In Table 3, a significant difference (p <0.05) was noted between the average number of TKR surgeries performed in the 1^st^, 7^th^, and 12^th^ lunar months versus the baseline month. There was no significant difference (p> 0.05) in PFF surgeries in the 7^th^ and 12^th^ lunar months versus the baseline month, but there was a significant increase in the 1^st^ lunar month. With this result, we further analyzed the patients separately by location of residence or gender.

**Table 3:** Mixed model analysis of the average number of surgeries categorized by the type of surgery and patients’ location of residence.

Surgery type	Location	Factor	F^†^	p-value
Total knee replacement	All	1^st^ lunar month^‡^	5.632	0.019*
		7^th^ lunar month	89.053	0.000*
		12^th^ lunar month	134.554	0.000*
	Urban	Gender^¶^	13.447	0.001*
		1^st^ lunar month	9.188	0.003*
		7^th^ lunar month	80.218	0.000*
		12^th^ lunar month	74.447	0.000*
	Rural	Gender	2.906	0.093
		1^st^ lunar month	2.928	0.093
		7^th^ lunar month	41.225	0.000*
		12^th^ lunar month	206.612	0.000*
Surgery for proximal femur fracture	All	1^st^ lunar month	16.042	0.000*
		7^th^ lunar month	0.172	0.680
		12^th^ lunar month	2.323	0.134
	Urban	Gender	0.111	0.740
		1^st^ lunar month	4.164	0.053
		7^th^ lunar month	0.887	0.354
		12^th^ lunar month	2.035	0.167
	Rural	Gender	0.012	0.913
		1^st^ lunar month	16.091	0.000*
		7^th^ lunar month	0.040	0.843
		12^th^ lunar month	0.506	0.484

The average number of surgeries performed in the 2^nd^, 3^rd^, 4^th^, 5^th^, 6^th^, 8^th^, 9^th^, 10^th^, and 11^th^ lunar months is set as baseline to which the average numbers of surgeries in the 1^st^, 7^th^, and 12^th^ lunar months are compared to, respectively.

^‡^‘1^st^ lunar month’ denotes the difference of number of surgeries between the 1^st^ lunar month and the baseline month.

^¶^Gender denotes the difference in numbers of surgeries between male and female patients.

^†^F value denotes the statistic for testing whether the average of numbers of surgeries are equal between compared groups.

* Level of statistical significance is set at p<0.05.

### Dips in TKR surgeries in Ghost Month and Chinese New Year

The analyses of TKR surgeries as sorted by location of residence were shown in Table 3. For urban patients undergoing TKR surgeries, there was a significant difference between male and female patients (p<0.05). The average number of TKR surgeries performed was significantly decreased in the 1^st^, 7^th^, and 12^th^ lunar months in urban areas and in the 7^th^ and 12^th^ lunar months in rural areas (p<0.05).

The analyses of the number of TKR surgeries sorted by gender were presented in Table 4. A significant difference in the number of TKR surgeries performed between urban and rural patients was observed in male (p<0.05), but not in female patients. Lower number of TKR surgeries was observed in 7^th^ and 12^th^ lunar months in male patients, and in 1^st^, 7^th^, and 12^th^ lunar months in female patients (p<0.05).

**Table 4:** Mixed model analysis of the average number of surgery categorized by the type of surgery and patients’ genders.

Surgery type	Gender	Factor	F^†^	p-value
Total knee replacement	Male	Location^¶^	9.284	0.003*
		1^st^ lunar month	0.048	0.828
		7^th^ lunar month	16.565	0.000*
		12^th^ lunar month	94.658	0.000*
	Female	Location	0.559	0.457
		1^st^ lunar month^‡^	15.830	0.000*
		7^th^ lunar month	106.442	0.000*
		12^th^ lunar month	169.826	0.000*
Surgery for proximal femur fracture	Male	Location	0.640	0.427
		1^st^ lunar month	12.218	0.002*
		7^th^ lunar month	0.050	0.824
		12^th^ lunar month	0.291	0.595
	Female	Location	1.905	0.173
		1^st^ lunar month	4.720	0.039*
		7^th^ lunar month	0.850	0.364
		12^th^ lunar month	2.799	0.107

The average number of surgeries performed in the 2^nd^, 3^rd^, 4^th^, 5^th^, 6^th^, 8^th^, 9^th^, 10^th^, and 11^th^ lunar months is set as baseline to which the average numbers of surgeries in the 1^st^, 7^th^, and 12^th^ lunar months are compared to, respectively.

^¶^Location denotes the difference of numbers of surgeries between urban and rural patients.

^‡^‘1^st^ lunar month’ denotes the difference of number of surgeries between the 1^st^ lunar month and the baseline month.

^†^F value denotes the statistic for testing whether the average of numbers of surgeries are equal between compared groups.

*Level of statistical significance is set at p<0.05.

### PFF surgeries not influenced in Ghost Month and lunar December

In both urban and rural patients, no significant differences were noted between genders in the number of PFF surgeries performed (p>0.05) (Table 3). Furthermore, there was no significant difference in the number of PFF surgery performed in the 1^st^, 7^th^ and 12^th^ lunar months in urban areas and in the 7^th^ and 12^th^ lunar months in rural areas as compared with the baseline month (p>0.05).

Regardless of gender, the number of PFF surgeries performed did not differ amongst those living in urban or rural areas (p>0.05) (Table 4). Compared to the baseline month, there were no statistical differences in the numbers of PFF surgeries in the 7^th^ and 12^th^ lunar month in either genders (p>0.05); however, it was higher in the 1^st^ lunar month in both genders (p<0.05).

## Discussions

### Cultural beliefs affect healthcare-seeking behaviors

Cultural beliefs, which permeate every aspect of health behavior, are known to influence healthcare-seeking behavior and surgery decision [15–17]. Culturally specific healthcare-seeking behaviors are significantly influenced by sociocultural factors [2, 3, 18]. Cultural beliefs and superstitions are known to affect behavior in every culture in different ways [19]. For example, in Ireland, some patients refuse to be discharged from the hospital on a Saturday [7, 20]. Friday the 13^th^ is deemed an unlucky day by most people in the Western World [5, 19]. A widespread and often heard belief in German-speaking countries is that lunar phase has an effect on surgery and a full moon has the most negative effects on surgical outcome [21, 22]. Chinese and Japanese consider the number of”four” unlucky because the words “death” and “four” are phonated almost identically in Mandarin and Japanese [23, 24]. Some Chinese hospitals intentionally skip the number “four” when numerating the floor and wards [8].

### Influence of lunar festivals on Taiwanese patients

In Taiwan, the Chinese New Year festival lasts for one month [25]. Like Thanksgiving, it is an important time for Taiwanese when we gather for the annual family reunion. According to tradition, the Chinese New Year is a time to honor the ancestors and deities. Most Taiwanese believe that it will be bad luck to begin a new year with an illness that requires surgery; therefore, people usually avoid elective surgery during this period.

The 7^th^ lunar month is the so-called Ghost Month in Taiwanese society [25]. It is believed that hungry and vicious ghosts spend the month seeking lost souls to take their place in the underworld. In Taiwanese traditions, rivers and lakes are believed to be associated with drowning accidents, mountainous areas are associated with monsters and ghosts and hospitals are where many spend their final hours, so Taiwanese avoid potentially dangerous activities such as watersports, mountain climbing, and undergoing surgeries during the Ghost Month to prevent possible encounters with ghosts. Lin et al showed that the belief of the Ghost Month is associated with reduced rates of caesarean-section deliveries among Taiwanese [9], and Huang et al showed that 45.1% of women who underwent a caesarean-section chose to do so to ensure astrologically auspicious time of delivery [26].

Although there are studies investigating the association between the taboos of the Ghost Month and the reduced rates of caesarean-section delivery, there are very few studies probing the influence of cultural taboos of the Ghost Month and the Chinese New Year on the numbers of elective surgeries and emergent surgeries. Our study is the first report to investigate this issue by using the nationwide and population-based database.

### Impact of Ghost Month and Chinese New Year on TKR surgeries

Regardless of location of residence or gender, there were significant decreases in average numbers of TKR surgeries in the 7^th^ and 12th lunar months. The difference may have resulted from the elective nature of TKR surgery. The lack of urgency with elective surgeries and the fact that postponing treatment does not increase the surgical complications, patients avoid undergoing surgery in the Ghost Month and the Chinese New Year due to cultural taboos.

In Taiwanese culture, men and women are of different social status in society. As patients were sorted by genders, a significant decrease in number of TKR in the 1^st^ lunar month was noted among female patients, but not in male patients. According to Taiwanese traditions, the chores and preparations leading up to the main ceremonial is done by women during the Chinese New Year [27]. The elderly women are usually in charge of preparing the reunion dinner, the ancestor worship, and other celebrated events. This may explain why the number of TKR surgery in female patients was significantly deceased in this lunar month.

### Impact of Ghost Month and Chinese New Year on PFF surgeries

Although there was a significant decrease in the number of TKR surgery performed in the Ghost Month, the 12^th^ and 1^st^ lunar month as compared to the baseline month, the emergent surgery such as PFF surgery was not influenced. These results reflect the emergent nature of PFF surgery. The delay of PFF surgery may extend the patient's hospital stay and increase complications. This result emphasizes the concept that clinical concern about patients’ safety overrides the superstitious belief of the Ghost month and the Chinese New Year taboos. Interestingly, we observed that the number of PFF surgery was significantly increased in the 1^st^ lunar month in patients of both genders. We believe this is the result of the increased travels during the 1^st^ lunar month.

### Limitations

These observed results in our study are based on Taiwan National Health Insurance Research Database. Due to the inherent limitation of the population-based data, there is a lack of detailed demographics about the physicians and the patients. Our research showed a statistical association between sociocultural beliefs and healthcare-seeking behaviors, but we were unable to conclude that the cause is purely due to cultural beliefs. Further studies are required to investigate other possible variables.

In Taiwan, the celebration of the Ghost Month lasts for a whole month; however, people do not have public holidays. The dip in numbers of TKR surgery in the Ghost Month should not be contributed to the impact of holidays. Though the Chinese New Year festival also lasts for a whole month, there are only five-day public holidays from the Lunar New Year’s Eve to the 4^th^ day of lunar New Year; therefore, the impact of holidays should be limited. Furthermore, it should be mentioned that most people undergoing TKR surgery are elderly. Whether they are retired or still work is unclear; therefore, further study is needed to investigate whether the decreased number of TKR surgery during Chinese New Year festival is due to cultural beliefs or cultural beliefs mixed with the impact of holidays.

Although Taiwanese consider it a taboo to undergo surgeries in the Ghost month or during the Chinese New Year, this issue about the quality of the surgeries performed in this time period has never been discussed. Further studies on surgical infection, morbidity, or mortality rate in the 1^st^, 7^th^, and 12^th^ lunar months are mandatory to clarify the safety of surgery in these months. [21, 22]

From the viewpoint of human resource, the manager of the hospital can adjust the personnel resources of nursing staff and other medical personnel accordingly during the Ghost month and the Chinese New Year. The government may take the responsibility to implement the civil education about the safety of operations performed during the Ghost months and the Chinese New Year festival.

## Supporting information

S1 Table The numbers of surgeries performed in each lunar month from 2000 to 2011. (DOCX)

S2 Table Mixed model comparative analysis of the average number of surgery between each lunar month as the patients categorized according to location of residence and genders. (DOCX)
