# Supplementary material for: The sociocultural effects on orthopedic surgeries in Taiwan
Source: PLoS One. 2018 Mar 29;13(3):e0195183. doi: 10.1371/journal.pone.0195183 (PMC5875864; doi:10.1371/journal.pone.0195183)
Supplement: S1 Table — (DOCX) [file pone.0195183.s001.docx]

**S1 Table. The numbers of surgeries performed in each lunar month from 2000 to 2011.**

| year | lunar  month | TKR  Male  all | TKR  male  urban | TKR  Male  rural | TKR  Female  all | TKR  Female  urban | TKR  Female  rural | PFF  Male  all | PFF  Male  Urban | PFF  Male  rural | PFF  Female  all | PFF  Female  urban | PFF  Female  rural |
| --- | --- | --- | --- | --- | --- | --- | --- | --- | --- | --- | --- | --- | --- |
| 2000 | 1 | 6 | 3 | 3 | 19 | 7 | 12 | 22 | 10 | 12 | 19 | 7 | 12 |
| 2000 | 2 | 2 | 1 | 1 | 16 | 8 | 8 | 26 | 13 | 13 | 18 | 10 | 8 |
| 2000 | 3 | 5 | 1 | 4 | 29 | 14 | 15 | 18 | 11 | 7 | 14 | 8 | 6 |
| 2000 | 4 | 6 | 5 | 1 | 26 | 13 | 13 | 15 | 4 | 11 | 9 | 4 | 5 |
| 2000 | 5 | 4 | 2 | 2 | 20 | 10 | 10 | 19 | 7 | 12 | 18 | 11 | 7 |
| 2000 | 6 | 10 | 4 | 6 | 23 | 9 | 14 | 11 | 7 | 4 | 7 | 5 | 2 |
| 2000 | 7 | 9 | 3 | 6 | 25 | 12 | 13 | 15 | 9 | 6 | 15 | 4 | 11 |
| 2000 | 8 | 13 | 5 | 8 | 20 | 8 | 12 | 19 | 9 | 10 | 15 | 5 | 10 |
| 2000 | 9 | 11 | 4 | 7 | 31 | 14 | 17 | 11 | 5 | 6 | 15 | 7 | 8 |
| 2000 | 10 | 12 | 2 | 10 | 19 | 8 | 11 | 20 | 10 | 10 | 13 | 6 | 7 |
| 2000 | 11 | 12 | 5 | 7 | 23 | 12 | 11 | 13 | 6 | 7 | 14 | 7 | 7 |
| 2000 | 12 | 7 | 2 | 5 | 14 | 7 | 7 | 27 | 12 | 15 | 20 | 7 | 13 |
| 2001 | 1 | 12 | 4 | 8 | 24 | 9 | 15 | 38 | 15 | 23 | 37 | 17 | 20 |
| 2001 | 2 | 7 | 3 | 4 | 25 | 15 | 10 | 22 | 10 | 12 | 21 | 12 | 9 |
| 2001 | 3 | 8 | 3 | 5 | 29 | 12 | 17 | 7 | 4 | 3 | 16 | 6 | 10 |
| 2001 | 4 | 9 | 3.5 | 5.5 | 27 | 11.5 | 15.5 | 16.5 | 9 | 7.5 | 20 | 8.5 | 11.5 |
| 2001 | 5 | 12 | 2 | 10 | 19 | 7 | 12 | 17 | 10 | 7 | 20 | 7 | 13 |
| 2001 | 6 | 11 | 6 | 5 | 31 | 10 | 21 | 18 | 4 | 14 | 11 | 3 | 8 |
| 2001 | 7 | 9 | 3 | 6 | 6 | 3 | 3 | 12 | 8 | 4 | 14 | 7 | 7 |
| 2001 | 8 | 10 | 4 | 6 | 20 | 9 | 11 | 20 | 9 | 11 | 23 | 10 | 13 |
| 2001 | 9 | 6 | 1 | 5 | 28 | 11 | 17 | 12 | 6 | 6 | 14 | 7 | 7 |
| 2001 | 10 | 20 | 8 | 12 | 41 | 21 | 20 | 27 | 10 | 17 | 20 | 9 | 11 |
| 2001 | 11 | 7 | 2 | 5 | 19 | 7 | 12 | 21 | 11 | 10 | 18 | 7 | 11 |
| 2001 | 12 | 6 | 4 | 2 | 9 | 6 | 3 | 10 | 4 | 6 | 10 | 3 | 7 |
| 2002 | 1 | 8 | 1 | 7 | 42 | 18 | 24 | 22 | 7 | 15 | 15 | 5 | 10 |
| 2002 | 2 | 10 | 3 | 7 | 43 | 20 | 23 | 21 | 11 | 10 | 15 | 7 | 8 |
| 2002 | 3 | 21 | 9 | 12 | 42 | 15 | 27 | 16 | 9 | 7 | 25 | 10 | 15 |
| 2002 | 4 | 10 | 3 | 7 | 27 | 14 | 13 | 10 | 7 | 3 | 18 | 9 | 9 |
| 2002 | 5 | 14 | 2 | 12 | 27 | 9 | 18 | 28 | 15 | 13 | 19 | 7 | 12 |
| 2002 | 6 | 16 | 9 | 7 | 42 | 20 | 22 | 19 | 8 | 11 | 16 | 12 | 4 |
| 2002 | 7 | 10 | 5 | 5 | 10 | 3 | 7 | 18 | 10 | 8 | 19 | 9 | 10 |
| 2002 | 8 | 7 | 3 | 4 | 35 | 16 | 19 | 17 | 10 | 7 | 16 | 8 | 8 |
| 2002 | 9 | 11 | 5 | 6 | 30 | 18 | 12 | 16 | 8 | 8 | 17 | 5 | 12 |
| 2002 | 10 | 7 | 4 | 3 | 20 | 11 | 9 | 16 | 5 | 11 | 15 | 7 | 8 |
| 2002 | 11 | 11 | 3 | 8 | 29 | 16 | 13 | 12 | 7 | 5 | 17 | 8 | 9 |
| 2002 | 12 | 3 | 1 | 2 | 20 | 9 | 11 | 24 | 8 | 16 | 14 | 10 | 4 |
| 2003 | 1 | 9 | 6 | 3 | 29 | 13 | 16 | 18 | 5 | 13 | 24 | 9 | 15 |
| 2003 | 2 | 16 | 8 | 8 | 29 | 13 | 16 | 23 | 12 | 11 | 21 | 11 | 10 |
| 2003 | 3 | 10 | 4 | 6 | 19 | 5 | 14 | 24 | 12 | 12 | 15 | 9 | 6 |
| 2003 | 4 | 2 | 0 | 2 | 3 | 1 | 2 | 16 | 11 | 5 | 19 | 8 | 11 |
| 2003 | 5 | 4 | 1 | 3 | 14 | 8 | 6 | 18 | 9 | 9 | 21 | 11 | 10 |
| 2003 | 6 | 16 | 4 | 12 | 34 | 14 | 20 | 17 | 10 | 7 | 20 | 11 | 9 |
| 2003 | 7 | 9 | 5 | 4 | 19 | 6 | 13 | 24 | 9 | 15 | 22 | 11 | 11 |
| 2003 | 8 | 7 | 3 | 4 | 32 | 16 | 16 | 26 | 9 | 17 | 23 | 9 | 14 |
| 2003 | 9 | 10 | 5 | 5 | 23 | 10 | 13 | 21 | 12 | 9 | 25 | 14 | 11 |
| 2003 | 10 | 11 | 6 | 5 | 24 | 11 | 13 | 26 | 11 | 15 | 23 | 11 | 12 |
| 2003 | 11 | 11 | 3 | 8 | 28 | 14 | 14 | 18 | 10 | 8 | 24 | 15 | 9 |
| 2003 | 12 | 5 | 1 | 4 | 8 | 3 | 5 | 16 | 8 | 8 | 23 | 10 | 13 |
| 2004 | 1 | 13 | 4 | 9 | 25 | 11 | 14 | 49 | 28 | 21 | 31 | 18 | 13 |
| 2004 | 2 | 7 | 1.5 | 5.5 | 38 | 17 | 21 | 20 | 11.5 | 8.5 | 19 | 8 | 11 |
| 2004 | 3 | 11 | 2 | 9 | 39 | 17 | 22 | 17 | 7 | 10 | 19 | 10 | 9 |
| 2004 | 4 | 15 | 6 | 9 | 38 | 16 | 22 | 25 | 11 | 14 | 19 | 6 | 13 |
| 2004 | 5 | 5 | 4 | 1 | 34 | 9 | 25 | 20 | 11 | 9 | 19 | 7 | 12 |
| 2004 | 6 | 10 | 4 | 6 | 36 | 16 | 20 | 24 | 11 | 13 | 21 | 10 | 11 |
| 2004 | 7 | 12 | 4 | 8 | 20 | 12 | 8 | 14 | 8 | 6 | 24 | 12 | 12 |
| 2004 | 8 | 10 | 7 | 3 | 40 | 16 | 24 | 20 | 11 | 9 | 13 | 4 | 9 |
| 2004 | 9 | 12 | 7 | 5 | 31 | 11 | 20 | 22 | 8 | 14 | 15 | 9 | 6 |
| 2004 | 10 | 8 | 3 | 5 | 37 | 17 | 20 | 17 | 11 | 6 | 26 | 11 | 15 |
| 2004 | 11 | 3 | 3 | 0 | 26 | 11 | 15 | 19 | 9 | 10 | 23 | 11 | 12 |
| 2004 | 12 | 3 | 2 | 1 | 6 | 3 | 3 | 14 | 6 | 8 | 20 | 9 | 11 |
| 2005 | 1 | 15 | 5 | 10 | 27 | 12 | 15 | 15 | 6 | 9 | 18 | 7 | 11 |
| 2005 | 2 | 8 | 5 | 3 | 32 | 17 | 15 | 28 | 14 | 14 | 31 | 16 | 15 |
| 2005 | 3 | 20 | 11 | 9 | 36 | 14 | 22 | 15 | 7 | 8 | 21 | 13 | 8 |
| 2005 | 4 | 6 | 2 | 4 | 33 | 14 | 19 | 17 | 10 | 7 | 28 | 5 | 23 |
| 2005 | 5 | 10 | 2 | 8 | 40 | 16 | 24 | 22 | 14 | 8 | 16 | 8 | 8 |
| 2005 | 6 | 6 | 1 | 5 | 47 | 20 | 27 | 19 | 7 | 12 | 21 | 3 | 18 |
| 2005 | 7 | 10 | 3 | 7 | 18 | 6 | 12 | 21 | 11 | 10 | 23 | 10 | 13 |
| 2005 | 8 | 12 | 5 | 7 | 42 | 13 | 29 | 15 | 5 | 10 | 18 | 9 | 9 |
| 2005 | 9 | 7 | 3 | 4 | 31 | 14 | 17 | 15 | 8 | 7 | 17 | 2 | 15 |
| 2005 | 10 | 11 | 4 | 7 | 37 | 16 | 21 | 22 | 5 | 17 | 22 | 10 | 12 |
| 2005 | 11 | 11 | 4 | 7 | 37 | 17 | 20 | 28 | 12 | 16 | 27 | 14 | 13 |
| 2005 | 12 | 6 | 1 | 5 | 15 | 7 | 8 | 28 | 10 | 18 | 18 | 9 | 9 |
| 2006 | 1 | 17 | 7 | 10 | 34 | 17 | 17 | 40 | 14 | 26 | 36 | 21 | 15 |
| 2006 | 2 | 14 | 6 | 8 | 37 | 18 | 19 | 19 | 5 | 14 | 20 | 11 | 9 |
| 2006 | 3 | 17 | 4 | 13 | 51 | 26 | 25 | 17 | 6 | 11 | 24 | 10 | 14 |
| 2006 | 4 | 11 | 2 | 9 | 23 | 12 | 11 | 12 | 7 | 5 | 17 | 7 | 10 |
| 2006 | 5 | 8 | 4 | 4 | 34 | 18 | 16 | 20 | 10 | 10 | 28 | 10 | 18 |
| 2006 | 6 | 19 | 5 | 14 | 43 | 21 | 22 | 15 | 6 | 9 | 21 | 11 | 10 |
| 2006 | 7 | 12.5 | 4.5 | 8 | 35.5 | 15 | 20.5 | 15.5 | 8 | 7.5 | 25 | 9.5 | 15.5 |
| 2006 | 8 | 10 | 2 | 8 | 39 | 15 | 24 | 23 | 10 | 13 | 22 | 10 | 12 |
| 2006 | 9 | 16 | 5 | 11 | 38 | 20 | 18 | 21 | 12 | 9 | 16 | 9 | 7 |
| 2006 | 10 | 12 | 6 | 6 | 38 | 13 | 25 | 31 | 15 | 16 | 14 | 4 | 10 |
| 2006 | 11 | 9 | 6 | 3 | 40 | 17 | 23 | 20 | 7 | 13 | 30 | 13 | 17 |
| 2006 | 12 | 3 | 0 | 3 | 9 | 4 | 5 | 7 | 6 | 1 | 13 | 6 | 7 |
| 2007 | 1 | 10 | 4 | 6 | 27 | 14 | 13 | 20 | 8 | 12 | 20 | 7 | 13 |
| 2007 | 2 | 24 | 10 | 14 | 42 | 20 | 22 | 21 | 7 | 14 | 33 | 11 | 22 |
| 2007 | 3 | 17 | 8 | 9 | 56 | 24 | 32 | 20 | 7 | 13 | 26 | 6 | 20 |
| 2007 | 4 | 19 | 16 | 3 | 50 | 19 | 31 | 25 | 16 | 9 | 27 | 13 | 14 |
| 2007 | 5 | 15 | 10 | 5 | 53 | 23 | 30 | 8 | 5 | 3 | 21 | 11 | 10 |
| 2007 | 6 | 19 | 12 | 7 | 60 | 29 | 31 | 16 | 7 | 9 | 25 | 9 | 16 |
| 2007 | 7 | 4 | 1 | 3 | 19 | 10 | 9 | 22 | 7 | 15 | 25 | 8 | 17 |
| 2007 | 8 | 19 | 12 | 7 | 63 | 24 | 39 | 18 | 9 | 9 | 23 | 12 | 11 |
| 2007 | 9 | 26 | 10 | 16 | 56 | 29 | 27 | 20 | 14 | 6 | 29 | 11 | 18 |
| 2007 | 10 | 12 | 3 | 9 | 49 | 23 | 26 | 24 | 8 | 16 | 28 | 10 | 18 |
| 2007 | 11 | 14 | 6 | 8 | 41 | 22 | 19 | 26 | 11 | 15 | 26 | 7 | 19 |
| 2007 | 12 | 5 | 2 | 3 | 24 | 17 | 7 | 17 | 7 | 10 | 27 | 7 | 20 |
| 2008 | 1 | 19 | 8 | 11 | 37 | 17 | 20 | 25 | 12 | 13 | 19 | 10 | 9 |
| 2008 | 2 | 14 | 4 | 10 | 44 | 16 | 28 | 21 | 12 | 9 | 19 | 10 | 9 |
| 2008 | 3 | 17 | 9 | 8 | 55 | 28 | 27 | 20 | 13 | 7 | 16 | 7 | 9 |
| 2008 | 4 | 18 | 7 | 11 | 72 | 35 | 37 | 24 | 10 | 14 | 26 | 15 | 11 |
| 2008 | 5 | 17 | 7 | 10 | 52 | 25 | 27 | 23 | 11 | 12 | 19 | 7 | 12 |
| 2008 | 6 | 13 | 7 | 6 | 69 | 33 | 36 | 23 | 9 | 14 | 20 | 8 | 12 |
| 2008 | 7 | 7 | 4 | 3 | 30 | 13 | 17 | 27 | 11 | 16 | 24 | 17 | 7 |
| 2008 | 8 | 22 | 9 | 13 | 36 | 21 | 15 | 14 | 10 | 4 | 21 | 14 | 7 |
| 2008 | 9 | 20 | 8 | 12 | 50 | 17 | 33 | 23 | 9 | 14 | 15 | 4 | 11 |
| 2008 | 10 | 26 | 9 | 17 | 52 | 24 | 28 | 19 | 8 | 11 | 27 | 10 | 17 |
| 2008 | 11 | 7 | 4 | 3 | 38 | 14 | 24 | 24 | 12 | 12 | 25 | 8 | 17 |
| 2008 | 12 | 8 | 3 | 5 | 20 | 13 | 7 | 37 | 22 | 15 | 37 | 19 | 18 |
| 2009 | 1 | 21 | 7 | 14 | 53 | 24 | 29 | 26 | 13 | 13 | 43 | 22 | 21 |
| 2009 | 2 | 19 | 10 | 9 | 71 | 37 | 34 | 11 | 4 | 7 | 21 | 8 | 13 |
| 2009 | 3 | 13 | 5 | 8 | 53 | 21 | 32 | 27 | 15 | 12 | 21 | 15 | 6 |
| 2009 | 4 | 9 | 6 | 3 | 50 | 28 | 22 | 23 | 15 | 8 | 12 | 8 | 4 |
| 2009 | 5 | 17 | 8 | 9 | 58 | 33.5 | 24.5 | 16.5 | 9 | 7.5 | 23.5 | 13 | 10.5 |
| 2009 | 6 | 22 | 9 | 13 | 62 | 35 | 27 | 27 | 13 | 14 | 24 | 16 | 8 |
| 2009 | 7 | 17 | 8 | 9 | 35 | 14 | 21 | 17 | 9 | 8 | 22 | 12 | 10 |
| 2009 | 8 | 17 | 10 | 7 | 71 | 38 | 33 | 21 | 12 | 9 | 17 | 7 | 10 |
| 2009 | 9 | 22 | 6 | 16 | 53 | 24 | 29 | 22 | 13 | 9 | 24 | 16 | 8 |
| 2009 | 10 | 17 | 7 | 10 | 43 | 19 | 24 | 19 | 7 | 12 | 29 | 12 | 17 |
| 2009 | 11 | 14 | 9 | 5 | 45 | 20 | 25 | 16 | 5 | 11 | 38 | 20 | 18 |
| 2009 | 12 | 4 | 1 | 3 | 13 | 9 | 4 | 10 | 6 | 4 | 7 | 1 | 6 |
| 2010 | 1 | 16 | 5 | 11 | 40 | 23 | 17 | 33 | 17 | 16 | 26 | 13 | 13 |
| 2010 | 2 | 26 | 15 | 11 | 51 | 26 | 25 | 18 | 11 | 7 | 28 | 17 | 11 |
| 2010 | 3 | 15 | 7 | 8 | 74 | 31 | 43 | 18 | 8 | 10 | 13 | 7 | 6 |
| 2010 | 4 | 18 | 6 | 12 | 50 | 25 | 25 | 17 | 8 | 9 | 23 | 11 | 12 |
| 2010 | 5 | 17 | 8 | 9 | 57 | 31 | 26 | 17 | 9 | 8 | 14 | 7 | 7 |
| 2010 | 6 | 12 | 10 | 2 | 67 | 36 | 31 | 22 | 11 | 11 | 23 | 17 | 6 |
| 2010 | 7 | 9 | 4 | 5 | 26 | 15 | 11 | 25 | 14 | 11 | 22 | 11 | 11 |
| 2010 | 8 | 20 | 6 | 14 | 63 | 37 | 26 | 26 | 12 | 14 | 29 | 15 | 14 |
| 2010 | 9 | 13 | 8 | 5 | 35 | 16 | 19 | 21 | 9 | 12 | 27 | 9 | 18 |
| 2010 | 10 | 14 | 6 | 8 | 58 | 34 | 24 | 21 | 10 | 11 | 31 | 14 | 17 |
| 2010 | 11 | 17 | 9 | 8 | 59 | 32 | 27 | 25 | 11 | 14 | 34 | 16 | 18 |
| 2010 | 12 | 6 | 6 | 0 | 21 | 13 | 8 | 26 | 12 | 14 | 29 | 12 | 17 |
| 2011 | 1 | 17 | 7 | 10 | 41 | 16 | 25 | 25 | 13 | 12 | 25 | 13 | 12 |
| 2011 | 2 | 14 | 4 | 10 | 66 | 27 | 39 | 21 | 8 | 13 | 36 | 25 | 11 |
| 2011 | 3 | 17 | 7 | 10 | 59 | 29 | 30 | 27 | 10 | 17 | 20 | 11 | 9 |
| 2011 | 4 | 22 | 11 | 11 | 71 | 41 | 30 | 18 | 10 | 8 | 23 | 10 | 13 |
| 2011 | 5 | 19 | 12 | 7 | 65 | 30 | 35 | 23 | 8 | 15 | 26 | 14 | 12 |
| 2011 | 6 | 11 | 4 | 7 | 64 | 34 | 30 | 12 | 7 | 5 | 29 | 19 | 10 |
| 2011 | 7 | 10 | 2 | 8 | 42 | 17 | 25 | 20 | 15 | 5 | 28 | 15 | 13 |
| 2011 | 8 | 20 | 9 | 11 | 64 | 41 | 23 | 17 | 9 | 8 | 21 | 16 | 5 |
| 2011 | 9 | 22 | 10 | 12 | 58 | 27 | 31 | 19 | 7 | 12 | 29 | 12 | 17 |
| 2011 | 10 | 17 | 10 | 7 | 45 | 23 | 22 | 18 | 12 | 6 | 22 | 11 | 11 |
| 2011 | 11 | 20 | 6 | 14 | 62 | 29 | 33 | 19 | 10 | 9 | 31 | 19 | 12 |
| 2011 | 12 | 1 | 1 | 0 | 0 | 0 | 0 | 3 | 2 | 1 | 3 | 2 | 1 |
