# Supplementary material for: The sociocultural effects on orthopedic surgeries in Taiwan
Source: PLoS One. 2018 Mar 29;13(3):e0195183. doi: 10.1371/journal.pone.0195183 (PMC5875864; doi:10.1371/journal.pone.0195183)
Supplement: S2 Table — (DOCX) [file pone.0195183.s002.docx]

**S1 Table. Mixed model comparative analysis of the average number of surgery between each lunar month as the patients categorized according to location of residence (Table A) and genders (Table B).**

**Table A.**

| Surgery type | Location | Factor | F | p-value |
| --- | --- | --- | --- | --- |
| Total knee replacement | Urban | Lunar month^a^ | 12.222^b^ | 0.000^a^ |
|  | Rural | Lunar month | 23.904 | 0.000 |
| Surgery for proximal femur fracture | Urban | Lunar month | 1.235 | 0.297 |
|  | Rural | Lunar month | 2.831 | 0.007 |

^a^‘Lunar month’ is a factor of which we compare the difference of average number of surgery between each lunar month.

^b^‘F’ is the statistic for testing whether the average numbers of surgery in each lunar month are equal; p-value < 0.05 means that they are not equal.

**Table B.**

| Surgery type | Gender | Factor | F | p-value |
| --- | --- | --- | --- | --- |
| Total knee replacement | Male | Lunar month^a^ | 9.912^b^ | 0.000 |
|  | Female | Lunar month | 24.271 | 0.000 |
| Surgery for proximal femur fracture | Male | Lunar month | 1.740 | 0.102 |
|  | Female | Lunar month | 1.904 | 0.061 |

^a^‘Lunar month’ is a factor of which we compare the difference of average number of surgery between each lunar month.

^b^‘F’ is the statistic for testing whether the average numbers of surgery in each lunar month are equal; p-value < 0.05 means that they are not equal.
